# Melanoma in the Eyes of Mechanobiology

**DOI:** 10.3389/fcell.2020.00054

**Published:** 2020-02-11

**Authors:** M. Manuela Brás, Manfred Radmacher, Susana R. Sousa, Pedro L. Granja

**Affiliations:** ^1^Instituto de Investigação e Inovação em Saúde, Universidade do Porto, Porto, Portugal; ^2^Instituto de Engenharia Biomédica, Universidade do Porto, Porto, Portugal; ^3^Faculdade de Engenharia, Universidade do Porto, Porto, Portugal; ^4^Institute for Biophysics, University of Bremen, Bremen, Germany; ^5^Instituto Superior de Engenharia do Porto, Instituto Politécnico do Porto, Porto, Portugal

**Keywords:** melanoma, mechanobiology, melanocytes, keratinocytes, invasion, adhesion, migration, metastization

## Abstract

Skin is the largest organ of the human body with several important functions that can be impaired by injury, genetic or chronic diseases. Among all skin diseases, melanoma is one of the most severe, which can lead to death, due to metastization. Mechanotransduction has a crucial role for motility, invasion, adhesion and metastization processes, since it deals with the response of cells to physical forces. Signaling pathways are important to understand how physical cues produced or mediated by the Extracellular Matrix (ECM), affect healthy and tumor cells. During these processes, several molecules in the nucleus and cytoplasm are activated. Melanocytes, keratinocytes, fibroblasts and the ECM, play a crucial role in melanoma formation. This manuscript will address the synergy among melanocytes, keratinocytes, fibroblasts cells and the ECM considering their mechanical contribution and relevance in this disease. Mechanical properties of melanoma cells can also be influenced by pigmentation, which can be associated with changes in stiffness. Mechanical changes can be related with the adhesion, migration, or invasiveness potential of melanoma cells promoting a high metastization capacity of this cancer. Mechanosensing, mechanotransduction, and mechanoresponse will be highlighted with respect to the motility, invasion, adhesion and metastization in melanoma cancer.

## Melanoma

Melanoma is one of the most severe skin cancers in humans with a mortality rate of 80% (Hofschroer et al., 2017), due to the high resistance of this tumor to radiotherapy and chemotherapy (Uong and Zon, 2010). The resistance to those treatments is due to high number of mutations (approx. 75%) affecting specific genes, which lead to an increasing proliferation and survival (Kozar et al., 2019). Immunotherapy is being exploited and investigated, and can be grouped in five types: (i) targeted antibodies (Sanlorenzo et al., 2014; Herzberg and Fisher, 2016); (ii) adoptive cell therapy (Sanlorenzo et al., 2014); (iii) oncolytic virus therapy (Franklin et al., 2017); (iv) cancer vaccines (Tuettenberg et al., 2007; Rodriguez-Cerdeira et al., 2017); and (v) immunomodulators (Johnson et al., 2014; Faries, 2016; Cancer Research Institute, 2019). Chemotherapy and immunotherapy, can be combined (biochemotherapy) regarding the stage disease (Sanlorenzo et al., 2014; Kozar et al., 2019). The disease appears due to the malignant transformation of melanocytes located in the stratum basale of the epidermis although it is not clear how a melanocyte becomes malignant (Uong and Zon, 2010). Bandarchi et al. (2010) suggested that a combination of up and down regulation of factors in several molecular pathways, seem to be the mechanism of transformation of normal into malignant melanocytes. If the disease is detected in an early stage, there is a high probability of disease control, however, when metastization occurs, the 5 years life expectancy drops to 14%. Some epidemiological evidence related to melanoma incidences in white and dark skinned population (Bandarchi et al., 2013) was presented by the authors whereas Bradford (2009) presented epidemiological data about the total numbers of melanoma incidences in the world. Dark skin results in a lower incidence of skin cancer due to the protection of the increased epidermal melanin, which filters UV light twice as good as white skin of Caucasian people (Bradford, 2009). Malignant melanoma are diagnosed by the ABCD rule, where A stands for asymmetry, B for borders irregularity, C for color variation and D for diameter higher than 6 mm (Bandarchi et al., 2010). Melanoma shows three perceptible steps in tumor progression: (i) one only affecting the epidermis; (ii) a second one affecting both the epidermis and the superficial papillary of the dermis; and (iii) a third one affecting the epidermis, dermis and hypodermis (vertical growth melanoma) (Bandarchi et al., 2010). Haass et al. (2005) divided the process of melanoma development in the following steps: (i) the damage of adhesion between keratinocytes by down-regulation of E-cadherin, P-cadherin, desmoglein, and connexins, which is influenced by growth factors produced by fibroblasts or keratinocytes; (ii) the interaction of melanoma with fibroblasts and/or melanoma with melanoma cells normally not found in melanocytes, is up-regulated by MCAM and N-cadherin; and (iii) alteration of integrin expression, inducing the loss of basement membrane anchorage (Haass et al., 2005). According to the At Melanoma Foundation (AIM), melanoma develops in a similar way than other cancers: the DNA of a gene that controls cell division and proliferation is mutated. This damaged gene results in a lack of control of cell division and growth. This DNA mutation is passed to the daughter cell during division. Melanoma is originated from a melanocyte that experienced too many mutations growing in an abnormal way. In the case of melanoma, this DNA mutation is caused by an overexposure to UV radiation, affecting the melanocytes (At Melanoma Foundation, 2014). When there is DNA damage, several genes may be affected, resulting in encoding errors of several molecules hampering their function (At Melanoma Foundation, 2014; Kunz, 2014) and there is a risk for a melanocyte to generate a melanoma. If the melanoma is not suppressed or treated, it will spread along the epidermis, after penetrating the dermis and hypodermis, getting in contact with the lymph and the blood vessels (At Melanoma Foundation, 2014). The main function of melanocytes is to produce melanin (responsible for skin pigmentation) that absorb UV light to avoid keratinocytes’ DNA damage (Hirobe, 2014). Keratinocytes will distribute melanin to the upper parts of the skin layer (Wang et al., 2017). Wang et al. (2017) also mentioned that hormonal factors, family history and cosmetics are the three main triggering and aggravating factors for melanoma development. When a primary melanoma is formed, it activates some signals, which will provide conditions that make the primary tumor strong such as: (i) attachment to the wall of the blood and/or lymphatic vessels, enabling the movement through a new organ; (ii) enough nutrients to grow; and (iii) it is resistant to the immune system. With the three conditions mentioned before the primary tumor has the capacity to promote the metastization (At Melanoma Foundation, 2014). Why can melanoma develop in some body parts that are not exposed to sunlight? It was suggested that there are different changes occurring in genes of tissue cells exposed, compared with the changes of genes of tissue cells not exposed to UV light. These mutations are transmitted into the next generations. Some genes appear during melanoma development, being not genetic. The transformation of melanocytes into melanoma cells is a very complex process, which involves several signaling pathways reviewed by Paluncic et al. (2016). Briefly, melanocytes are formed from their precursors: melanoblasts. Melanoblasts differentiate into melanocytes, which get mature and start melanin production on melanosomes, which will be transferred to keratinocytes. Melanomagenesis depends on the microenvironment, genetic and environmental factors. Although genetic factors are crucial, they are not sufficient to induce melanomagenesis (Paluncic et al., 2016). Melanoma tumors are different from patient to patient. The environment, where melanoma tumors grow, is very complex depending on the interactions with ECM, microvasculature, fibroblastic cells and changing concentration of growth factor, cytokines, and nutrients like glucose, oxygen, etc (Paluncic et al., 2016). Some signaling pathways are more involved in tumor development, whereas other signals are more crucial in the metastization process. The description of biochemical pathways signaling is beyond of this review, however the readers can find more information in different sources (Dissanayake et al., 2007; Straussman et al., 2012; Moriceau et al., 2015; Stark et al., 2015; Wang et al., 2016; Ahmed and Haass, 2018; Kodet et al., 2018). Melanoma can be pigmented or not pigmented. The color of melanoma is due to the existence of melanin, secreted and stored in melanosomes in the cytoplasm of melanocytes. There are two types of melanin: eumelanin (has a black color) and pheomelanin (has a red/yellow color). Normal melanocytes may or may not secret either type of melanin (Lassalle et al., 2003). Mechanical properties and cell pigmentation are correlated (Sarna et al., 2013, 2014).

As melanoma needs a high amount of iron and copper, the development of substances that target these ions could be another methodology (Gorodetsky et al., 1986).

## Mechanobiology of Skin Cells

Currently, mechanobiology brings together biologists and biophysicists in order to better understand the role of mechanical forces in cell motility, adhesion and invasion. For developing therapies that avoid the invasiveness of cancer cells, it is crucial to understand the mechanisms of tumor invasion. The mechanical stiffness of tumors is correlated with its invasiveness, which was demonstrated by a study with cancer ovarian cells: more invasion corresponds to softer cells resulting in deformation and shape changes, being suitable for metastization (Swaminathan et al., 2011). Makale (2007) raised three hypotheses correlating cancer and cell mechanobiology: (i) the cell biology of tumor invasion is a key to understand cancer treatment; (ii) cancer cells activate physiological mechanisms and migratory processes that are used by cells usually during embryo morphogenesis; and (iii) forces that are sensed by the tissue and cells trigger the differentiation and spreading of tumors. In fact, mechanical forces are generated by tissues, being sensed by cells, during embryogenesis and tumor expansion (Makale, 2007).

In melanoma the Epithelial to Mesenchymal Transition (EMT) switching, and forces behind this process (Wei et al., 2015) are crucial processes regarding the loss of connections between melanocyte and keratinocytes; melanocytes acquired the embryonic phenotype, promoting cells invasion, their intravasation in body fluids and the extravasation to other organs. Arriving at their target destination, tumor cells are reverted to their original phenotype known as Mesenchymal to Epithelial Transition (MET), establishing secondary tumors. The production of metalloproteinases (MMP) will induce ECM degradation, favoring metastization (Paluncic et al., 2016). Migration of collective cells interact with the extracellular environment via integrins (Hegerfeldt et al., 2002). When normal cells are compressed because of a tumor, these forces will be sensed by cell body, through membrane molecules, the integrins, which are connected to the cytoskeleton, and to the nucleus. All cells respond to these external forces organizing cellular components in the cell and generating biochemical signals that will help them to adapt to external physical pressure. The normal ECM produced by fibroblasts also induces pressure on tumor cells via its cytoskeleton, resulting in cell and nucleus deformation. A consequence of nucleus compression could be the modulation of gene expression that may induce changes in cell viability, promoting locomotion, due to the adoption of a more malignant phenotype. In spite of high tumor stroma stiffness, tumor cells are deformable and have some degree of plasticity (Makale, 2007). In melanoma, the relation among melanocytes, keratinocytes, fibroblasts and the ECM mediate and control this disease. In the next paragraphs, mechanobiology of these cell types and ECM contribution to melanoma will be addressed.

### The Role of Melanocytes

**FIGURE 1:**
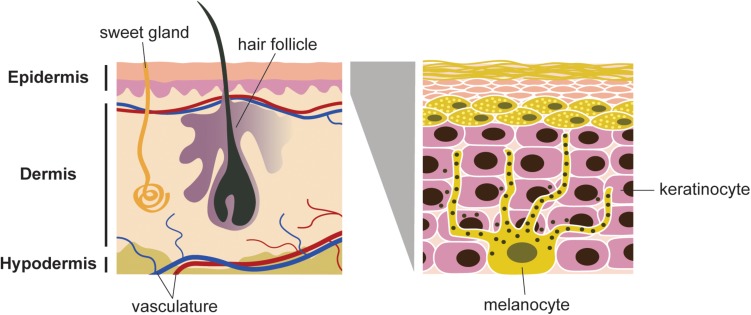
Schematic representation of skin and cell population. Melanocytes are located in the basal layer of epidermis. Each melanocyte is connected to several keratinocytes. Melanosomes (black spots) are distributed in the dentrites, produce and store melanin. The dendrites allow the transfer of the melanin to keratinocytes, which will be transferred to the stratum corneum layer of the epidermis.

Melanocytes are located in the basal layer of the epidermis, eyes, ears, hair and meninges (Bandarchi et al., 2013). Melanocytes have a dendritic shape (Figure 1). They are originated from migratory embryonic cells, called neural crest cells, which have a high capacity of migration (Uong and Zon, 2010). Melanocytes are the cells responsible for skin pigmentation, through the production of melanin, also named melanogenesis (Lassalle et al., 2003). They are present in the epidermis and exhibit long dendritic protrusions, which are extended through the epidermal keratinocytes. Melanocytes synthesize melanosomes and transport them to the keratinocytes (Zhang et al., 2004).

Melanocytes adhere to keratinocytes through E-cadherins, Desmoglein (DSg)/Desmocollin (DSc), Connexins (CX) molecules (Li et al., 2003) and to the basement membrane through integrins (Wang et al., 2016). The ratio of melanocytes and keratinocytes is around 1:36/1:40. 8 to 12% of malignant melanoma are associated with mutations on CDKN2A (cyclin-dependent kinase Inhibitor 2) gene (Bandarchi et al., 2013). Uong and Zon (2010) also mentioned that the pathways involved in melanocyte development and proliferation start to be subverted in melanoma formation. BRAF and NRAS (a gene of the RAS family identified for the first time at human neuroblastoma) (Pugh et al., 2013) overactive mutations are found in 65 and 20% of melanomas, respectively. CDKN2A is lacking by melanoma cells, consequently the encoding suppressor genes (INK4a and ARF) are not active (Bandarchi et al., 2010).

Malignant melanocytes are characterized by changes of biophysical properties (like shape, elasticity and stiffness) which will contribute to the cell locomotion, invasion and metastization in other organs. In this case, the plasticity of melanocytes is very important, since they need to be highly deformable. Since the mechanical properties of melanoma cells may be affected by the production of melanin, this has to be considered (Sarna et al., 2013). Melanin and melanogenesis may act in two different directions: melanin act as a protection shield to radiation, however it also attenuates the effectiveness of radiotherapy, chemotherapy and phototherapy in the case of melanotic melanoma. This characteristic can provide tumor growth and progression (Slominski et al., 2015).

AFM was used to observe the shape of human epidermis melanocytes in culture and to observe the melanosome transfer *in vivo*. Filopodia originated from the dendrite tips of the cell body of melanocytes, which contained melanosomes have been observed (Zhang et al., 2004). Van Den Bossche et al. (2006) hypothesized different mechanisms for melanin transfer: (i) keratinocytes phagocyte the melanocyte dendrites; (ii) the melanosomes release melanin into the extracellular space by melanosome exocytosis, being internalized by the keratinocytes; (iii) melanosomes are transported via membrane nanotubes; and (iv) the melanocytes enriched of melanosomes are phagocytosed by the keratinocytes (Van Den Bossche et al., 2006).

Wu et al. (2012) studied melanocytes of mice lacking myosin Va (Myo5a). This protein is involved in the accumulation of melanosomes in the melanocyte dendrites. When myosin Va is present, melanosomes stay concentrated in the centre of cancerous melanocytes reducing melanosome transfer to keratinocytes (Wu et al., 2012). Wäster et al. (2016) studied the relation between UV skin radiation and skin pigmentation response. They concluded that when pigmented skin is exposed to UVA, there is a spillage of extracellular vesicles from the melanocyte’s plasma membrane. This does not happen when pigmented skin is exposed to UVB (Wäster et al., 2016).

AFM was used to study the morphological changes of melanocytes exposed to the melanocyte stimulated hormone α-MSH, which induces melanization (Shin et al., 2014). The authors found that the roughness inside the pore-like structures on dendrites increased and MSH produced an increase in the size and density of the melanosomes. However, regarding the response to the presence of α-MSH, more studies are needed to correlate morphological shape alterations with the movement of melanosomes (Shin et al., 2014). Human melanocyte’s pigmentation in melanoma cells are also important in regulation of elastic properties according to Choi et al. (2014). The authors studied the effect of substrates with different stiffness on the pigmentation, using normal human melanocytes (NHM) and melanoma cells (MNT1) cultured on polydimethylsiloxane (PDMS) coated with laminin. They observed a reduction of dendrite formation in both cell type as well as a difference on the transference rate of melanosomes for both cell type on softer substrates. Cells cultured on stiffer substrates showed lower migration capacity once traction forces formation are more evident through via focal adhesion integrin complexes (Choi et al., 2014).

Based on some studies of cell elasticity, cancer cells typically show a decrease in stiffness relatively to normal cells. Sarna et al. (2013) described a study of elastic properties comparing melanoma cells and healthy melanocytes. Basically, the authors used AFM to determine the stiffness of human melanoma cells SKMEL-188 with different stages of pigmentation and compared with SKMEL-188 cells not pigmented and with NHMs healthy human melanocytes (Sarna et al., 2013). They found that healthy melanocytes were softer than cancer cells. This feature could be related with the amount of melanin present in melanoma cells, resulting in some limitations for cancer diagnosis using the stiffness. Lazova and Pawelek (2009) mentioned that melanoma cells do not excrete the pigment (Lazova and Pawelek, 2009) like healthy melanocytes do, which may result in pigment accumulation in melanoma cells in relation to melanocytes. This could explain the higher stiffness of cancer cells, since they are often highly pigmented (Lazova and Pawelek, 2009). In conclusion, by AFM nanoindentation assays it is not possible to distinguish melanoma cells from melanocytes. AFM can be used as a complementary technique, however, this study was useful in understanding better pigmented cell elasticity. Sarna et al. (2014) also mentioned the need to understand if there is a correlation between the pigmentation of melanoma cells and the metastization phenotype process. In this process, malignant cells must pass the epithelial barrier, having the capacity to deform their cell body, including the nucleus to migrate through tissue. Cells with low Young’s moduli exhibit a great potential to invade tissue (Sarna et al., 2014), so this is why biophysical properties, as cell elasticity, must be taken into account. The authors showed that melanin granules inhibit the transmigration potentialities of melanoma cells. For non-pigmented melanoma cells, the stiffness was lower, and the transmigration efficiency was higher. This study contributed with new insights about the relationship between pigmentation and elasticity of melanoma cells in terms of migration and invasion.

Some substances are responsible for the retraction of melanocyte dendrites. Ito et al. (2006) have studied the effect of dendrite retraction using a flavonoid substance. In this case, TYRP-1 signaling pathway is interrupted, once this molecule is important for melanosome migration. Rho signaling molecule, when activated induces dendritic retraction, reducing the melanosome transfer to the keratinocytes but does not affect the amount of melanin production. However, this melanin will not be transferred due to the dendrite’s retraction. The mechanism of dendrite retraction via RhoA involves changes in cytoskeleton and microtubules organization due to actin and tubulin-β disassembly. These changes will induce stress fiber formation promoting actin polymerization and reorganization of microtubules (Ito et al., 2006). Rac is involved in melanocyte dendrite growing, inducing the appearing of lamellipodia (Scott, 2002). The dendrite tips of melanocytes are also stimulated by the α-MSH and endothelin – 1 proteins from keratinocytes that are surrounding the melanocytes and the ECM produced by the keratinocyte (Kippenberger et al., 1998). Kippenberger et al. (1998) demonstrated that co-cultures of melanocytes and keratinocytes in the presence of high concentration of calcium, showed sheets of keratinocytes formation, surrounding by melanocytes with polar dendrites extended around the differentiated keratinocytes. Differentiated keratinocytes will have stable areas of contact with melanocytes due to the calcium gradient. The normal melanocytes that lose their dendrites soon will be in an apoptosis state (Kippenberger et al., 1998). The senescence of primary melanocytes could also be important in the melanoma appearance (Feuerer et al., 2019), however, there are no studies that correlate melanocytes’ senescence with mechanical properties measured by AFM. Some studies showed a correlation between the melanocytes aging and senescence biomarkers (Wang and Dreesen, 2018) and exposition of melanocytes to light (Bennet et al., 2017). AFM is a powerful tool to assess the morphological and elastic properties regarding the interface between primary melanocytes and primary melanoma with and without senescence. In opposition to the melanoma cell lines that are immortalized and thus, submitted to several passages, the primary melanocytes and primary melanoma cells should be assessed in order to evaluate what passage the senescence will not interfere with mechanical properties. The quantification of senescence level can be performed using the quantification of the Population Double Level (PDL) method (Kruse and Patterson, 1973).

### The Role of Keratinocytes

Keratinocytes are the predominant cell type in the epidermis, most of them (90%) being located at the outmost epidermis layer (McGrath et al., 2004). Keratinocytes are connected to each other through actin cell junctions (Hsu et al., 2018).

The main function of keratinocytes is to form a barrier against pathogenic microorganisms, heat, UV radiation and water loss. They also produce cytokines that attract leukocytes to the location of pathogen invasion (Hall et al., 2014). Keratinocytes also produce different keratins. Keratins (type 1, 5, 10, and 14) are the main structural protein of their cytoskeleton, all of which play an important role in the mechanics of these cells (Ramms et al., 2013). Keratins become altered when responding to stresses, resulting in its modification and reorganization (Karantza, 2011). Keratinocytes become corneocytes, when they are completely differentiated, losing their nucleus and cytoplasmic organelles (Mi, 2009). Differentiation of keratinocytes is driven by a calcium gradient, which is established between the interior of the cell and the ECM (Proksch et al., 2008) and regulated by vitamin D3 (Bikle, 2004), cathepsin E (Kawakubo et al., 2011), TALE homeodomain transcription factors (Jackson et al., 2011), and hydrocortisone (Rheinwald and Green, 1975). Keratinocytes are affected by senescence, losing their biological function with age (Orioli and Dellambra, 2018). Keratin, actin filaments and microtubules form the cytoskeleton network of keratinocytes, providing shape and structure of cells allowing transmission of mechanical loads (Silver and Siperko, 2003). For keratinocytes migration one of the most important parameters is the substrate stiffness (Ramms et al., 2013), since it is expected that the keratinocytes must change their shape during migration through the epidermis until they reach the outmost layer and become corneocytes (Zarkoob et al., 2015). This change in shape will affect also the components existing inside the cytoplasm, namely the cytoskeleton, in which keratin fibers are crucial (Ramms et al., 2013). Ramms et al. (2013) used AFM to probe keratinocytes (in the nuclear region and in the cell body) deficient in keratins finding that they are softer than healthy ones. In addition, with magnetic tweezers, the viscous contribution to the displacement of magnetic beads increased, corroborating the results achieved by AFM. These authors also found that the softening was reversible and could depend on the re-expression of two types of Keratins (K4 and K15) only (Ramms et al., 2013). Bar et al. (2014) reported the skin fragility in mice and hampered desmosomes adhesion when there is a lack of all keratins Bar et al., 2014. AFM showed a reduction of adhesive forces and membrane stability in murine keratinocytes when lacking keratin filaments (Vielmuth et al., 2018). Adherent junctions (AJ) have functions in mechanosensing and transducing mechanical forces between the plasma membrane and the actomyosin cytoskeleton. Desmosomes and intermediate filaments promote the mechanical stability to maintain tissue architecture and integrity when they are submitted to mechanical stress. Both (AJ and desmosomes) connect the actin and keratin filament network of adjacent cells. Desmosomes are important for intercellular cohesion, where keratins determine cell mechanics but are not involved in tension generation (Hatzfeld et al., 2017). Zarkoob et al. (2015) investigated the role of substrate stiffness on keratinocyte colony formation *(in vitro*) during epithelial formation, due to calcium exchange during keratinocyte cell culture. Keratinocytes cultured on soft polyacrylamide substrates (*E* = 1.2 KPa) presented small contact areas, an increase in migration velocity and in colony formation compared to those cultured on stiff polyacrylamide gels (*E* = 24 KPa) (Zarkoob et al., 2015). How melanoma cells escape the keratinocyte’s control invading other tissues and form metastasis, is still unclear. In a healthy tissue, there is a homeostasis maintained by each cell. In this normal tissue, keratinocytes are the “surveyors” of melanocytes, controlling their growth and behavior through complex paracrine growth factors and cell-cell adhesion molecules. When alterations occur to this homeostatic balance, cell-cell adhesion and cell communication molecules change and induce melanoma development resulting in the damage of the epidermal melanin unit, inducing the continuous melanocyte proliferation, and eventually leading to melanoma development (Haass et al., 2005). Chung et al. (2011) suggested keratinocytes-derived ECM factors may act as regulators of melanocytes. The authors showed that laminin-332, a component of basement membrane, plays a crucial role in the adhesion and migration of melanocytes and melanoma. It is known that keratinocytes regulate the behavior of melanocytes such as proliferation, melanin synthesis and dendrite formation and the ECM also regulates different cell behaviors (Chung et al., 2011).

### The Role of Fibroblasts and the ECM

Fibroblasts are produced in the bone marrow. They synthesize the components of the ECM, for instance collagen, glycosaminoglycans and elastic fibers. Fibroblasts also produce a structural network called “stroma” in animal tissues (connective tissue), playing an important role in cancer and in wound healing (Desjardins-Park et al., 2018). Kwa et al. (2019) mentioned that cancer is associated with fibroblasts and how these fibroblasts affect metastization. Fibroblasts produce high amounts of ECM molecules, cytokines and growth factors contributing to the stroma, cancer progression and metastization due to several pathways. These pathways could be a target for new cancer treatment strategies to be developed. However, it is difficult to characterize this subtype of fibroblasts and to identify specific molecules involved in this process (Kwa et al., 2019). Mechanosensing has a large impact in Cancer-Associated Fibroblasts (CAFs). Fibroblasts sense mechanical stresses and activate mechanosignaling processes, which use mechanoreceptors located in the cell membrane, the cytoskeleton and transcription factors. The ECM stiffness is determinant in the mechanosignaling, which by itself can activate the mechanosensing pathways in CAFs, leading to ECM production and stiffness increasing, supporting different CAFs differentiation (Kwa et al., 2019).

**FIGURE 2:**
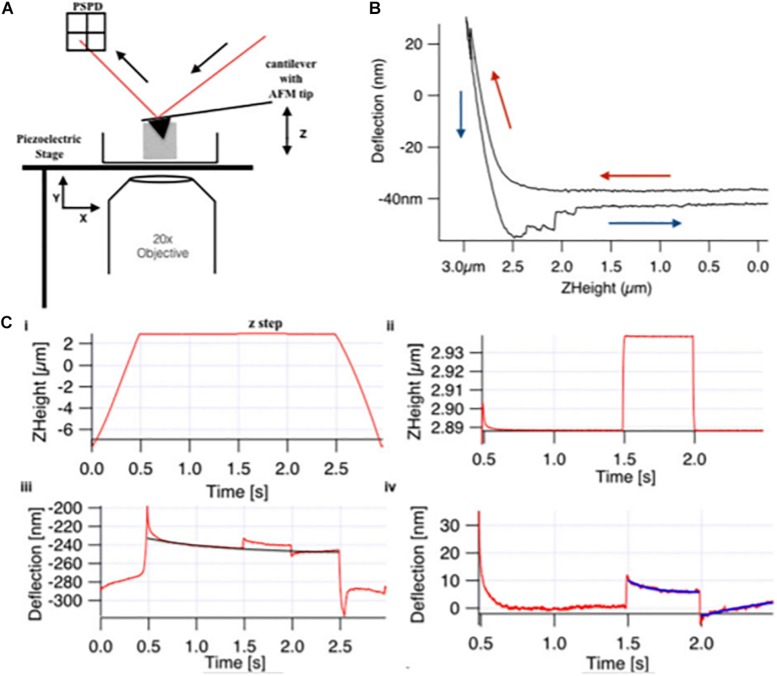
Basic components of **(A)** AFM and **(B)** force curve with **(C)** viscoelastic creep response measurement. **(A)** The basic four components of AFM **(i)** a laser diode, **(ii)** a cantilever of 0.01 N/m spring constant with 30 nm radius AFM pyramidal tip, **(iii)** a position-sensitive photo detector (PSPD), and **(iv)** xyz-piezo stage. **(B)** Sample indentation by the AFM tip obtains the force curve that gives the approach (red arrow) and retract (blue arrow) curve on deflection vs. Z-height graph, and apparent Young modulus was calculated by applying Hertz model to the approach curve. In creep response curve **(C)**, the Z-height profile **(i)** shows the approach and retract ramp toward the cell for 3 s, and in-between, there is a z step, which is applied at *t* = 1.5, which is enlarged in panel **(ii)**. **(iii)** The deflection data show global creep of the cell, which includes the creep after loading and unloading step, which is enlarged in panel **(iv)**, and global creep was determined by the exponential fit (black curve) and was subtracted for qualitative analysis (reproduced with permission from John Wiley and Sons with Copyright Clearance Center’s Right, Journal of Molecular Recognition, 2018) (Viji Babu et al., 2018).

The study of mechanosignaling and fibroblasts response to the ECM stiffness was conducted using hydrogels, to mimic pathologically the ECM of tissues. Viji Babu et al. (2018) have used the AFM to study the stiffness and viscoelasticity of normal, scar and Dupuytren’s disease fibroblasts, growing on top of soft (1 KPa) and stiff (50 KPa) hydrogels. Basically, a MLCT-Bio pyramidal cantilever (nominal spring constant of 0.01 N/m, Bruker, United States) was used to indent the fibroblasts, using the z-step response methodology, in which the elasticity and viscosity was assessed (Figure 2). The authors wanted to evaluate the effect of TGF-α in the fibroblast’s elasticity. The authors concluded that Dupuytren’s fibroblasts increased their elastic moduli and became stiffer in TGF-β1 presence, whereas they did not find significant changes in the elastic moduli of scar and normal fibroblasts, before and after addition of TGF-β1. Dupuytren’s fibroblasts presented a wide number of well-organized stress fibers and bundles of stress fibers were even thicker in presence of TGF-β1 (Viji Babu et al., 2018).

RhoA signaling is the key for the fibroblast’s reaction to ECM stiffness. RhoA is activated by a stiff matrix, causing cell contraction and actin polymerization, inducing the differentiation of fibroblasts in myofibroblasts. Therefore, CAFs need the activation of this pathway, since it is required for their contractile and invasive properties. It has been mentioned that there are several CAF subtypes, however, there is a lack of information about their plasticity, which could be important for the progression and metastization of cancer (Kwa et al., 2019). AFM could give new insights regarding the characterization of fibroblasts subtype based in their morphometric characteristics (shape factor, volume and area) and mechanical properties. As far as we know, there is not any specific study in literature, which could be useful for the development of therapies directed to a specific fibroblast subtype related with melanoma. This study could also promote new insights to develop therapeutic strategies to other cancer types.

The ECM is composed by (i) the structural proteins collagen and elastin; (ii) adhesion proteins such as fibrillin, fibronectin, vitronectin and laminin; and (iii) the proteoglycans (which have a protein core bound to glycosaminoglycans) (Makale, 2007). The ECM is responsible for cellular support. Variations in its structure and composition alter the mechanical properties of the ECM. Generally, the external forces applied to cell-matrix complexes are transmitted to the interior of the cell by integrins according to three steps (Roca-Cusachs et al., 2009): (i) integrins binding to ECM molecules; (ii) transmission of forces to the interior of the cells, through integrins, where they are transformed into biochemical signals (mechanotransduction); and (iii) integrins binding to cytoskeleton, transmitting the forces all over the cell, and reinforcing their adhesion properties to resist the force. The integrins inside of cell recruit several other molecules, which bind to the cytoskeleton and/or signaling proteins to form what is called “focal adhesion” to resist to the external force (Roca-Cusachs et al., 2009).

What are the mechanical cues sensed by cells when forces are applied from the outside? Integrin-associated complexes (IACs) in cellular membranes establish the connection between the ECM and the cytoskeleton of the cell, via actin filaments (Haase et al., 2016). Haase et al. (2016) found that physical forces stemming from the ECM have a huge impact on gene regulation; it has been hypothesized that gene expression is affected by nucleus deformation, which was observed in 3T3 fibroblasts. The actin and microtubule network generates nuclear deformation. The deformation of the nucleus will be anisotropic being observed along the shorter axis and is intrinsic to the nucleus. This behavior is due to the chromatin organization and lamin-A expression (Haase et al., 2016). The ECM uses the concept of “tensegrity,” which employs tensile forces and rigid support structures to distribute and manage the loads mediated by the cellular cytoskeleton complex. In the cytoskeleton microfilaments and microtubules, which are connected to the ECM, there are forces in between (Makale, 2007). This means that the pressure exerted on the ECM will influence the cell’s behavior in terms of differentiation, motility, adhesion, invasion, and metastasis (Makale, 2007). ECM mechanical properties are directly related with elastin fibers, collagens, glycosaminoglycans (GAGs) and proteoglycans. Elastin fibers contribute to cyclic stretching during life; collagen fibers contribute to the tissue stiffness and mechanical strength of tissues and GAGs affect ECM viscoelasticity, forming bridges between the collagen fibers and contributing to the compressive tissue stiffness. Cells sense the forces exerted by the ECM, converting these stimuli into intracellular signal pathway, which further downstream regulate transcriptional changes (Hsu et al., 2018). Several mechanosensors are identified in cells: glycocalyx, lipid raft/caveolin-1, cell adhesion structures (integrin, hemi-desmosomes and focal adhesion), ion channels (calcium-sensing receptor), transient receptor potential channels, connexins, intercellular complexes (desmosomes or cadherin) and the cytoskeleton (particularly the actin filaments) (Hsu et al., 2018). Any abnormality that causes changes in the ECM mechanical properties, cell interactions, or cellular response to mechanical stimuli will induce tensional homeostasis, contributing to several diseases like cancer and fibrosis (Hsu et al., 2018). Integrins (transmembrane proteins) are responsible for transmission of forces between ECM and the interior of the cell. Tumor cells expressed the avb3 integrin and the invasiveness of melanoma cells depends on the expression of β-3 and β-1 integrins, which are related with their aggressive properties (Petitclerc et al., 1999; Hegerfeldt et al., 2002). Therefore, one therapeutic strategy could contribute to the reduction of these integrins expression (Makale, 2007). Stiffness of tumors is important since it activates biochemical pathways regarding the cell cycle, EMT, cell motility and leads to compression in neighboring healthy cells. These healthy cells may then activate tumourogenic pathways (Broders-Bondon et al., 2018).

**FIGURE 3:**
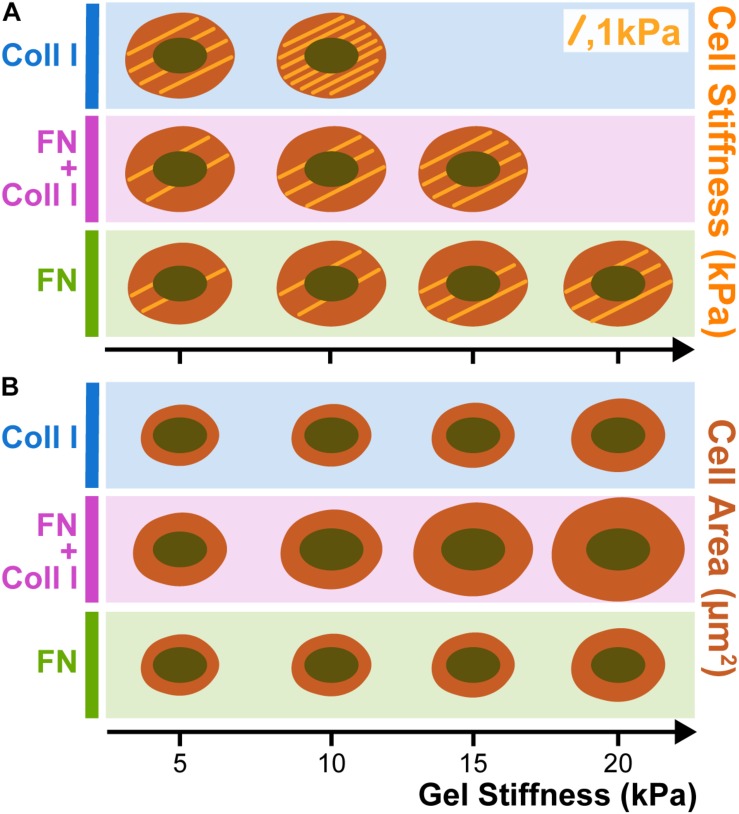
Melanoma stiffness **(A)** and spreading **(B)** in function of Collagen type I (Coll I) and Fibronectin (FN) presence in hydrogels. When A7 cells are plated on gels coated with saturating amounts of either FN or Coll I, they spread to approximately the same extent **(B)**, but the cells on Coll I are much stiffer than those adherent to FN **(A)**. When both FN and Coll I are present, allowing both α1 and β3 integrins to bind, adherent area increases, but cell stiffness reaches an intermediate value between those found on FN or Coll I alone. Each trace drawing inside the cells corresponds to 1 KPa (based on Kandow et al., 2007).

Cell spreading depends on substrate stiffness, which, in turn, depends on the complex adhesion type. A7 melanoma cells were seeded in polyacrylamide gels laminated with collagen type I (Coll I), fibronectin (FN) and a mixture of Coll I and FN (Winer et al., 2011). Figure 3 shows the stiffening and spreading of filamin A-expressing A7 human melanoma cells and is compared with different stiffnesses substrates coated with FN or Coll I. Stiffening but not spreading of A7 melanoma cells depends very strongly on whether integrins specific for FN or Coll I are engaged. When A7 cells are plated on gels coated with saturating amounts of either FN or Coll I, they spread to approximately the same extent (Figure 3B), but the cells on Coll I are much stiffer than those adherent to FN (Figure 3A). When both FN and Coll I are present, allowing both α1 and β3 integrins to bind, adherent area increases, but cell stiffness reaches an intermediate value between those found on FN or Coll I alone (Kandow et al., 2007).

## Cell Migration and Progression in Melanoma

### Motility

**FIGURE 4:**
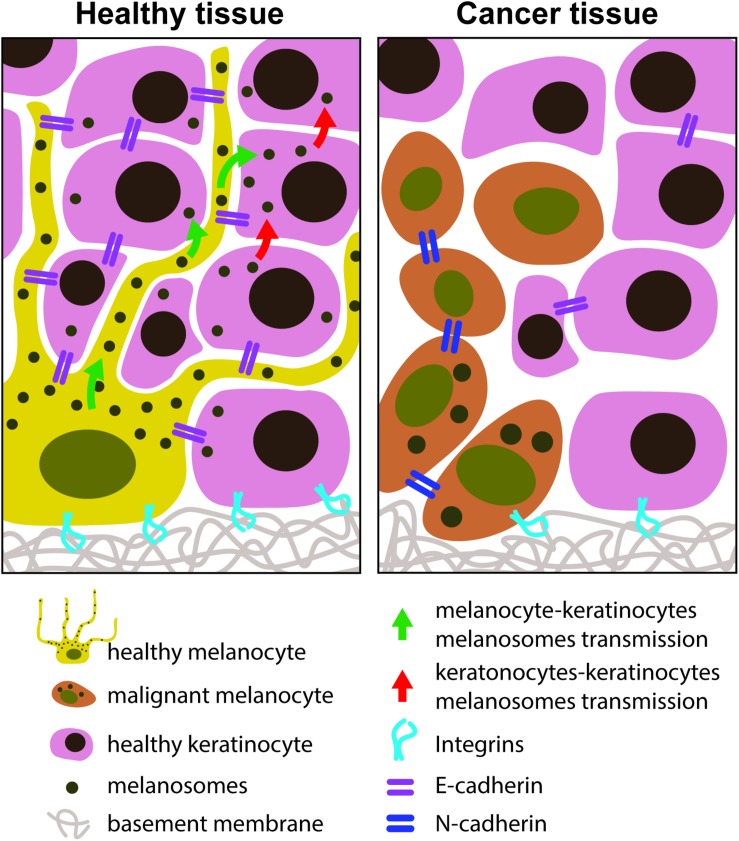
Melanocytes – keratinocytes adhesion molecules in healthy and in cancer tissues. In a healthy state the melanocyte-keratinocyte interactions are characterized by the existence of E-cadherin adherent junctions. The keratinocytes and melanocytes are connected to the ECM through the integrins. In a cancer state, the E-cadherins are lost and N-cadherins are the adherent junctions stablished between melanocytes. Melanocyte dendrites retracted and melanocytes acquired a round shape phenotype.

Melanoma starts with the lost binding intercellular connections between melanocytes and keratinocytes (Figure 4).

In the process of melanoma development, due to EMT, cells acquired a mesenchymal stem cell phenotype, contributing to cell migration (Bandarchi et al., 2010). The EMT model is very well explained by Nieto et al. (2016) and Brabletz et al. (2018), the forces involved in EMT and its relation with cancer metastasis are described by Wei et al. (2015). After losing their connections, melanocytes move due to a motility process.

**FIGURE 5:**
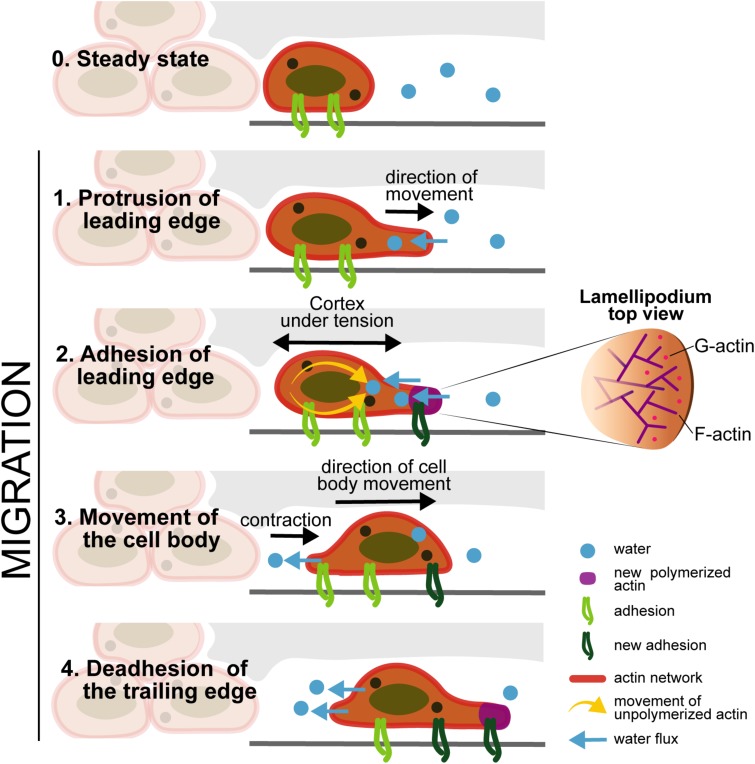
Cell motility: Stage 0 (Steady state) -Cells maintain the connections to the ECM through the integrins; Stage 1 (Protrusion of the leading edge) -internal forces induce the F-actin polymerization and the water flow enters through the cell membrane; the actin polymerization induce the formation of protrusions; Stage 2 (Adhesion of leading edge) - the cortex under tension, accumulates new actin, through the movement of unpolymerized actin to the protrusion edge, and new adhesion molecules appear in the protrusion part. Stage 3 (Movement of the cell body) - there is the contraction of the cell in the same direction of the cell movement, and the water flow exits from the cell in the opposite direction, contributing to cell motility; Stage 4 (Deadhesion of the trailing edge) - Deadhesion on the cell back edge: the integrins in the back of the cell decreases, and new adhesion molecules appeared in the front of the cell in the same direction of cell movement based on Tao et al. (2017).

Motility is the capacity of a living system to move independently, using metabolic energy to perform mechanical work and should not be confused with mobility (Allen, 1981; Figure 5). For cell migration, there is the contribution of the internal forces (which are generated by some structures present inside each cell, such as the cytoskeletal filaments) and the external forces (the shear flow of blood or lymphatic fluids contributions). The motility of cells through tissues is a process mediated by internal forces, which are related with the polarization of the cell body within the ECM. This motion depends on shape changes, cell-substrate interaction, and the generation of forces from the cells mediating through the tissue. The internal forces responsible for cell motility are composed by the following steps: (i) cytoskeletal polarization, forming extensions of actin filaments located in the leading edge; (ii) cell protrusions connect to the ECM scaffold by cell surface adhesion receptors; (iii) contraction of actomyosin motors that are connected with the ECM substrates exerting traction forces; (iv) bipolar tension, which is transmitted through the substrate, allows the retraction of the cell rear completing the movement of the cell; and (v) when cells move, they produce ECM degrading proteases and the transmigrated ECM is remodeled by proteolysis (Tao et al., 2017). Tao et al. (2017) also refers that the actin cytoskeleton exhibits viscous and elastic responses, which can be characterized by the relaxation time observed in a creep response after applying a stress. The relaxation corresponds to the time scale where the cell cytoskeleton can flow and behave in an elastic way. Cytoplasm is more than cytoskeleton: it has a lot of water content (around 70%), which also play a role in cell motion, since water flows in between the cytoplasm organelles. The external forces are related with the water flow and osmotic pressure, as well to the fluid shear stress of the blood and lymph. The water can pass through the cell membrane via aquaporin in both directions, so the cell can change its volume, either forced by external cues like the osmotic pressure difference between inside and outside of the cell or actively controlled by ion pumps (Tao et al., 2017).

Cytoskeleton rearrangement occurs through the arrangement of three proteins: Rho, Rac and Cdc42. The Rho GTPase family since they influence the appearance and structure of filopodia, stress fibers and adhesion plaques of melanoma cells (Tao et al., 2017) encourage actin retraction; Rac was treated to encourage lamellipodia extension but block actin depolymerization; Cdc42 was assumed to encourage filopodia elongation and block actin depolymerization (Tao et al., 2017). Motility is resulting from what is calling the mechanotransduction “inside-out” combining with a water flow in and out.

In human melanoma, by micropore filtration, it was found that vimentin and keratin intermediate filament expression results in a more migratory and invasive phenotype (Gao, 2014). The authors also found that damage of actin filaments and microtubules increases the deformability contributing to motility and metastization (Gao, 2014). Cell motion requires that the cytoplasm viscosity change in the cell periphery through the transformation of the gel state to a fluidic one. This mechanism also induces formation of protrusions that facilitate cell locomotion. Actin filaments are bond to the plasma membrane by a glycoprotein. In the presence of this glycoprotein cells are stable, the protrusions are well organized, occurring cell movement. If this glycoprotein is not present, as in the case of certain human melanoma, locomotion is impaired, and the plasma membrane shows blebbing (because of the cytoskeleton instability). If the presence of the glycoprotein is re-established, blebbing disappears and cell movement is recovered. This glycoprotein also increases the stiffness of normal cells, meaning that in melanoma cancer the lack of this protein increases the deformability and contributes to the efficiency of the migration process of tumor cells (Gao, 2014).

The whole cell, including the nucleus, must present a certain plasticity. Inside the nucleus, there is chromatin together with lamins in coordination with the cytoskeleton, maintaining shape and mechanical stability of the nucleus. Chromatin works like an elastic spring and is responsible for the force response to small deformations of the nucleus. Lamin A deforms easily for small extensions and gives stiffness to resist large nuclear deformations (Stephens et al., 2019). Microtubules in the cell cytoskeleton exert forces generated by dynein that can deform or even cause the rupture of the nucleus. Vimentin protects the nucleus giving it stability and perinuclear stiffness, which hinders 3D motility. Actin also stabilizes the nucleus shape together with microtubules (Stephens et al., 2019). Alterations to chromatin due to mechanical or physical properties, which could alter the nuclear shape, may be a biomarker of disease. Mechanotransduction pathways that sense and respond to ECM forces will regulate the shape of the nucleus. Physical properties of nuclear components also regulate cellular functions and could give rise to abnormal nuclear shape or other diseases (Stephens et al., 2019). In these cases, nuclear disruption may occur originating inflammation, senescence or cancer.

Wen et al. (2017) studied the morphological differences in four cell types: a human healthy melanocyte and melanoma cell types M14, A375 and MV3. Transwell migration assays were performed and filopodia, lamellipodia, stress fiber and adhesion plaque were characterized. Melanocytes did not show stress fibers or adhesion plaques. In contrast, the three melanoma cell types showed thin and short filopodia and stress fibers. The transwell test showed that melanocytes have no ability to migrate through the transmembrane, in contrast to the other three cell types (Wen et al., 2017). Rho GTPases family members are involved in several cellular activities (like stress fibers and adhesion plaques) through the signal transduction, such as cytoskeleton rearrangement, cell proliferation, cell polarization and cell chemotaxis, being reduced in melanoma cells. The overexpression of Rho can inhibit the expression of other factors like P21 (a tumor-inhibitor factor). Thus, Rho may be crucial for the inhibition of melanoma cells. Rac1 is responsible for microtubule stabilization, being increased in melanocytes (Wen et al., 2017).

### Invasion

**FIGURE 6:**
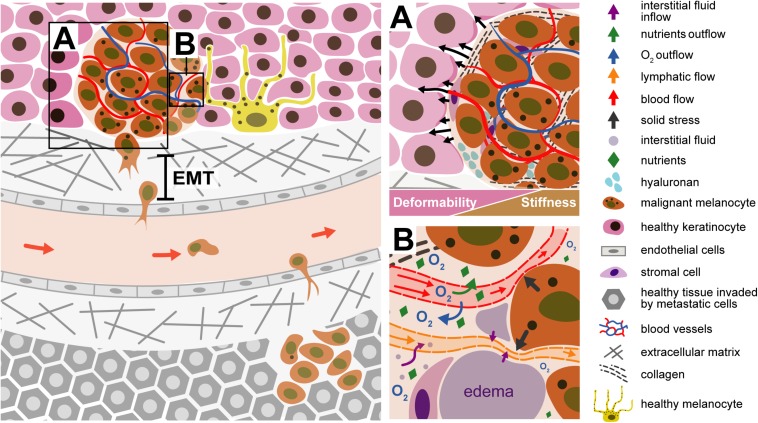
Illustration of a malignant melanocyte invasion to a new organ. **(A)** Tumor microenvironment - the tumor tissue becomes stiffer comparing with the surrounding tissue microenvironment; the generation forces (solid stress) by the tumor components contribute to displacement of the surrounding normal tissue. The EMT process contributes to cell movement. The malignant melanocyte, due to internal forces, is detached from the primary tumor through the ECM, passing through the endothelial cell of the blood or lymph vessel (intravasation). The tumor environment is stiffer than the healthy cells, being not so deformable, when compared with the tissue around. **(B)** Liquid stress is related to the forces exerted by the blood, lymphatic and interstitial vessels. The tumor compresses the vessels, which are squeezing; the O_2_, comes outside the vessels and the tumor is deprived of oxygenation, must develop conditions to grow in hypoxia. The compression of lymphatic vessels avoids the drainage of excessive interstitial liquids, resulting in accumulation of fluids in the interstitial space and formation of edema.

Weder et al. (2014) also demonstrated, by AFM studies, that the transformation of Radial Growth Phase (RGP) to Vertical Growth Phase (VGP) contribute to the melanoma progression. The transformation of RGP to VGP cells is associated with decrease of cell stiffness, which promotes the cell deformation and invasion into the surrounding stroma and the intravasation (Weder et al., 2014). Invasion is the capacity of cell penetration into neighboring tissues. This concept also includes the extravasation, however, this concept will be described in the section “Metastization.” The invasion of melanoma cells occurs through the blood or the lymphatic system. Primary melanoma will grow and spread horizontally on the top of skin layer (epidermis). When the melanoma cells go deeply into the dermis (vertical growth) they can reach the lymph nodes or blood vessels (intravasation). Some cells may get detached from the primary tumor and spread along the vessels, forming metastases, which eventually spread to regional lymph nodes (the cervical, the axillary and the inguinal basin). If the melanoma cells will be confined to these sentinel nodes, they can be removed by surgery. In the lymphatic vessels, there are the lymphocytes B and T and the Natural Killer cells. Melanoma cells must have a certain plasticity to cross not only the lymph vessels (endothelium tissue) but also the smooth muscle that is in the external part of the lymphatic vessels (this smooth muscle tissue avoids that the lymphatic fluid goes in the opposite direction as well as the valves that are inside of these vessels). The vessels are porous, allowing the entrance of other cells such as cancer cells (At Melanoma Foundation, 2014). The Circulating Tumor Cells (CTCs) are a subject that is transversal to several cancer types. Studying this cell type could be beneficial for developing new therapeutic strategies against metastization (Wang et al., 2018). CTCs traveling across the blood need to be strong enough to withstand the blood shear stress (Huang et al., 2018). Figure 6 illustrates different steps of melanoma progression, from invasion until the metastization process.

The mechanical forces exerted by the tumor components are named “solid stress.” These forces drive the tumor tissue and deforms the surrounding normal tissue. The normal tissue, in turn, resists tumor expansion. Solid stress affects tumor pathophysiology in two manners: directly by compressing cancer and stromal cells and indirectly by deforming blood and lymphatic vessels. Cell compression induces alterations in gene expression, cancer cell proliferation, apoptosis and invasiveness, stromal cell function, and the organization of extracellular matrix and its synthesis. Blood and lymphatic vessel compression reduces the delivery of oxygen, nutrients and drugs, creating a hypoxic and acidic microenvironment, compromising therapeutic outcomes (Padera et al., 2004; Stylianopoulos and Jain, 2013; Jain et al., 2014). The forces exerted by the fluid components in the tumor are called “fluid stress.” These forces include the microvascular and interstitial fluid pressures (MVP and IFP, respectively), as well as the shear stress exerted by blood and lymphatic flow on the vessel wall and by interstitial flow on cancer and stromal cells as well as extracellular matrix (Koumoutsakos et al., 2013). Fluid stresses are a combination of the effect of structure of tumor vessels and blood and lymphatic vessels compression. The compression of blood vessels reduces the cross section of vessel, increasing the resistance to the blood flow, which can affect the MVP, shear stress and perfusion (Jain, 1988). The compression of lymphatic vessels avoid the drainage of excessive interstitial liquids, resulting in accumulation of fluids in the interstitial space and formation edema (Jain and Baxter, 1988; Baxter and Jain, 1989). The hyper-permeability of the tumor vessels, formed during the process of angiogenesis, usually lead to increased fluid flux from the vascular to the interstitial space. This fluid leakage reduces tumor perfusion and, along with the loss of functional lymphatics through physical compression, elevates IFP. The elevated IFP reduces perfusion, because the upstream microvascular pressure is communicated to the extravascular space and then to downstream vessels, minimizing the pressure drop between upstream and downstream segments of vessels leading to reduced flow. The elevated IFP and MVC will contribute to a barrier to drug delivery therapies (Jain et al., 2014).

In the eyes of mechanotransduction, fluid shear stress activates YAP1 protein (yes-associated protein 1) which starts a step cascade that promotes cancer cell motility, since it is a potent oncogene present in several cancer types (Huang et al., 2005). Lee et al. (2017) showed that mechanical forces exerted by the fluid flow regulate cell behavior, being crucial for the metastasis process (Lee et al., 2017). Chivukula et al. (2015) also demonstrated that cancer prostate cells may be able to adapt to the fluid shear stress (FSS) (Chivukula et al., 2015). Micropipette aspiration was used to measure the Young’s modulus of prostate epithelial and prostate cancer cells. The Young’s modulus was determined before and after the exposition (low and high) to FSS. Normal prostate cells were 140% stiffer than cancer cells not exposed to FSS. After exposing cancer cells to low and high FSS, the authors obtained only 47 and 77% of the initial stiffness, respectively. The Young’s modulus of normal cells did not change significantly when in contact with FSS. Blood vessels are very diverse in terms of size, porosity, structure, cellular components, mechanical properties, depending on their location in the body and the specific function of the local environment. Endothelial cells can sense the strength of shear flow stresses and respond to it with the production of nitric oxide and prostacyclin to relax vascular smooth muscle cells, increasing the diameter of the vessel and thus decreasing the flow shear stress (Huang et al., 2018). When melanoma cells move through the blood or lymph, they are subjected to shear forces caused by liquid flow. How melanoma cells react to the shear stress of the circulating fluid? For blood fluid characterization, the following parameters must be taken into account: the pressure, the fluid shear stress, the viscosity, the blood vessels stiffness and the circulating blood cells. All maintain the mechanical equilibrium in a healthy state. If this equilibrium is lost, it will contribute to the appearance of hematological or vascular diseases (Qiu et al., 2019). Several techniques can be used to measure the mechanical properties of blood fluid, blood cells, and tissues in contact with blood and adherent cells to blood vessels. AFM (Kasas and Dietler, 2013), optical tweezers (Tan et al., 2012) and Micropipette aspiration (Daza et al., 2019) are used to measure the mechanical properties of cells. AFM is used to measure the mechanical properties of tissues (Lekka et al., 2012); the micro-viscometer is used to measure the viscosity of blood (Kim et al., 2017) and traction force microscopy is suitable to measure focal adhesion forces exerted by adherent cells to the ECM or other substrates (Checa et al., 2015). A detailed description of these techniques is beyond the scope of this manuscript.

Marsavela et al. (2018) mentioned the importance of the Melanoma Circulating Tumor Cells (MelCTCs) as a benefit and challenge for clinical applications. The MelCTCs cells are clones of primary and/or circulating metastatic cells in blood. MelCTCs can pass through the blood flow using a passive intravasation, which requires low energy. The melanoma cell detached from the tumor due to the increased blood flow and or the existing low levels of CDH1 (tumor suppressor gene). Activated intravasation occurs when some melanoma metastatic proteins (NEDD9 and DOC-3) are expressed by the cell, inducing the Rac18 activation, causing changes in actin assembly in the cell causing the migration into vessels. The intravasation by active process means better metastatic ability, since the melanoma cell survives in the blood stream (Mumford and Robertson, 2014).

In spite the MelCTCs being in low concentration in the periphery of blood system, to collect these cells is an advantage, mainly when the biopsy of the tumor is difficult to perform due to its localization. EpCAM, are found in cancers such as breast and prostate cancers but, are not present in melanoma cells, once the breast and prostate cancer cells are original from epithelia tissue and the melanocytes are derived from neural crest. Other limitation is that MelCTCs are a very heterogeneous population (Marsavela et al., 2018). There are several isolation techniques for MelCTCs, from the use of simple surface markers until new methodologies based in the physical characterization of MelCTCs. MelCTCs have a size range between 13 and 21 μm, what confirm the molecular and phenotype heterogeneity (Aya-Bonilla et al., 2017). More studies are needed to be performed in the MelCTCs to know more regarding its phenotype and know their behavior when subjected to some drug treatment. The authors hypothesized that AFM could have a significant contribution in this field regarding the study of morphometric parameters when the MelCTCs are subjected to specific drugs. Force spectroscopy molecular recognition studies could be helpful to improve the knowledge about the receptors of MelCTCs through the utilization of polyclonal antibodies in order to find receptors expressed in these cells. These results could lead to the development of markers and detection strategies helping in the isolation of these cells. The new findings could be promising to find new drugs in immunotherapy fields.

### Adhesion

Melanoma cell adhesion molecule (MCAM) is a cell-surface adhesion molecule expressed by melanocytes in over 70% of metastatic melanoma cells, not being expressed in normal melanocytes *in vivo* (Yoshioka et al., 2003). Endothelin-1 (ET-1) contributes to the up-regulation of MCAM that will contribute for the melanoma progression (Mangahas et al., 2004). Chen et al. (2019) mentioned that the MCAM is a driving force for the dissemination of melanoma cells through specific binding in original skin lesions (Chen et al., 2019). There are antigens shared between melanoma and vascular endothelial cells. The intravasation of the melanoma cells is based in melanoma adhesion molecules and the interaction with surface molecules on endothelial cells. L1-CAM from melanoma cells bind to integrin avb3 inducing intravasation. N-cadherin also bind to endothelial cell (Braeuer et al., 2013). Klemke et al. (2007) demonstrated that the interaction between α4β1 and the adhesion molecule VCAM-1 enhances the migration of human melanoma cells across activated endothelial cells. The transmigration through the endothelium of high metastatic human melanoma cell lines also occurred through the unstimulated VCAM-1 negative endothelial cells. The authors of this work used human melanoma cells that were devoid of β2 integrins, therefore did not bind to ICAM-1. The transmigration through the endothelial cells is mediated via interactions of other adhesion molecules than VCAM-1 or ICAM-1 (Klemke et al., 2007). Rebhun et al. (2010) also studied the importance of the integrin 4α binding existing in B16-F1 melanoma cells in the adhesion to the lymphatic vasculature, *in vitro* and how this mechanism impact the lymphatic metastization of melanoma cells *in vivo*, in mice. Lymphatic vessels express the VCAM-1 molecules in endothelial cells. The B16-F1 metastized in 30% in lymph nodes, while the B164α + metastized 80% in the lymph nodes; B164α- (deficient in 4α) was non-tumourigenic. This study demonstrated that 4α expressed in melanoma cells contributes to the metastization of the lymph nodes, due to a stable connection established between the melanoma cell and the lymphatic vasculature (Rebhun et al., 2010). Therapeutic strategies must be developed, avoiding these specific binding molecules. Using drugs that provide the capacity to compete and bind first to 4α, avoiding the VCAM-1 contact or a drug that works through the weakness forces stablishing between these binding complexes. Immunotherapy development could be based in this approach.

Kolasińska et al. (2019) also mentioned that sialic acid (SA) monosaccharides are commonly found at the outermost end of the sugar chains of various glycoconjugates. SA external position on cell surface molecules strongly influence cell behavior because SA may interact with other cell surface molecules and ECM proteins. SA has been reported to have an important role in the progression of melanoma cells. The ECM integrins also play an important role in melanoma cell adhesion to fibronectin (FN) as well as melanoma cell migration rate on FN. The main receptor of FN (α5β1) can be modified by the α2-3 and α2-6 linked SA residues and its expression level on melanoma cells was positively associated with the progression of melanoma (Kolasińska et al., 2019). The authors suggest that AFM molecular recognition force spectroscopy could be used to assess the unbinding forces between SA and α2-3 and α2-6 integrins. These findings could contribute to the development of new therapies that could avoid the bindings between the SA and the integrins.

### Metastization

**FIGURE 7:**
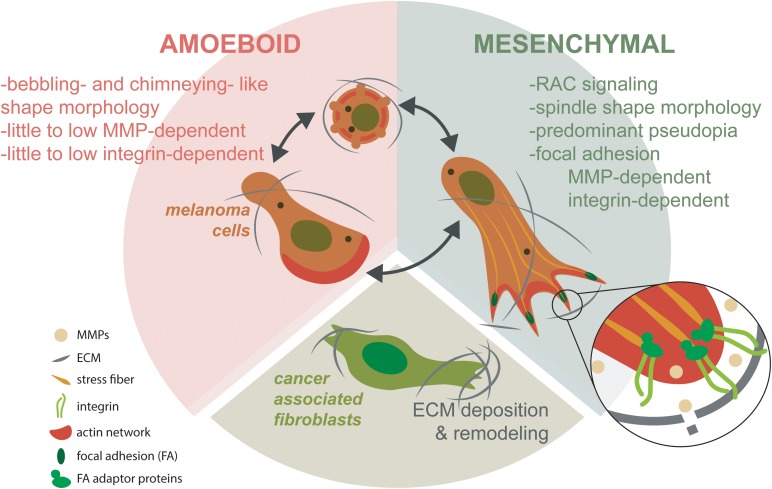
Mesenchymal and amoeboidal 3D migration dynamic modes in metastatic melanoma. Mesenchymal migration depends on RAC signaling to establish front-rear 3D polarity. This polarity generates a predominant pseudopodia, producing the characteristic spindle like morphology. Mesenchymal migration depends on MMP-dependent ECM degradation and integrin-dependent cell-matrix attachment protein complexes known as focal adhesions (FAs). Most probably, FAs are related as being important for exocytic trafficking of MMPs. In contrast, amoeboid migration assumes different migration modes from blebbing, chimneying to actin gliding modes. Amoeboidal migration requires little to no integrin activity and or MMP-mediated matrix degradation. CAFs facilitate ECM changes through deposition of fibronectin that crosslinks collagen I fibers. CAF-dependent contractility further in reorganization of ECM influencing melanoma migration and survival mechanosensory proteins (based in Ju et al., 2018).

The main target organs for melanoma metastases are the lungs, the liver and the brain (Ju et al., 2018). Metastization occurs after a cell/group of cells detached from the primary tumor, passed through the basal membrane, migrated through the ECM, entered the circulation system (blood and lymph nodes), adhered to epithelial cells, leaving again the circulatory system and entering finally to another organ(s), where the secondary tumor(s) establishes (Hofschroer et al., 2017). Contributing factors and associated physical phenomena can be observed and quantified during metastization. Like in other cancers (Kim et al., 2016), melanocyte(s) detached from the primary tumor must have high “strength” that make them different from those that stayed in the primary tumor. These characteristics are related with the nuclear size, architecture and cytoskeleton network. The physical processes associated are related to cell deformability, contractility, migration, traction forces and invasion. As previously referred, the malignant cells need to cross the basement membrane that is associated with structure integrity, being stiff and leakiness. Blood or lymph vessels contribute to the cell mobility because of their structure integrity, leakiness and osmolality changes. Associated physical processes like fluid shear stress, friction, and adhesion and cell deformation contribute to mobility of malignant melanocytes through the flow due to its nuclear architecture, nuclear size, cytoskeleton network and surface molecules. The ECM also contributes to the cell migration to a new organ(s) through the pore size and substrate properties. The physical phenomena associated are: matrix stiffness, adhesion and traction stresses and invasion (Kim et al., 2016). Melanoma cell invasion is facilitated by the relation between the melanoma, ECM and stroma, in which the mechanical ECM has an important role (Tanner, 2018). The tumor-stroma tissue involves extracellular matrices, fibroblasts, adipocytes, pericytes, endothelial, immune, and inflammatory cells, such as macrophages and neutrophils (Pietras and Ostman, 2010). Transformed CAFs have shown to facilitate tumor invasion through the integrin αVβ3-dependent fibronectin secretion, which promote the mechanical changes in the ECM through the contraction of collagen fibers (Gao et al., 2013; Kim et al., 2016). Ju et al. (2018) described the dynamic of 3D migration in metastatic melanoma. This process involves the mesenchymal and amoeboid migration (Figure 7). Mesenchymal process is based in a front-rear 3D polarity to generate a predominant pseudopodium, showing the spindle like shape and depending on the ECM degradation by the MMPs and integrin-dependent cell-matrix attachment protein complexes known as focal adhesions (FAs). These FAs work like exocytic structures for MMPs, mediated by the cortical microtubule stabilization complexes containing microtubule associated proteins. The amoeboid migration is based from blebbing, chimneying, to actin gliding modes. This last migration process requires little to no integrin activity and or MMP-mediated matrix degradation. Transformed cancer-associated fibroblasts (CAFs) are known to facilitate ECM changes through deposition of fibronectin that crosslinks collagen I fibers. CAFs - dependent contractility further in reorganization of ECM influencing melanoma migration and survival mechanosensory proteins (Ju et al., 2018).

According to Huang et al. (2018), the fluid shear stress (FSS) is important for migration and metastization. The shear stress flow is a variable in tumor metastasis-related fluid microenvironment. Blood shear stress levels (1–3000 dyn/cm^2^) is higher than interstitial flow (0.1 dyn/cm^2^) and lymph flow (0.64 dyn/cm^2^). In blood, only 0.02% of CTCs survive. In lymphatic fluid, the FSS generated at sentinel lymph nodes can significantly upregulate ICAM-1 in lymphoid endothelial cells, facilitating lymph node metastasis. Low FSS facilitate cell melanoma adhesion facilitating the extravasation and metastization (Huang et al., 2018). The MelCTCs are promising cancer biomarkers but isolation from the blood is very challenging because of their huge heterogeneity, which makes difficult their biological and clinical study (Aya-Bonilla et al., 2017). In melanoma, the metastization occurs in several organs being the lung, brain (Tawbi et al., 2018) and liver, the most affected ones (Damsky et al., 2010). “Why is melanoma so metastatic?” asked Braeuer et al. (2013). Melanoma cells are very efficient in invading the host immune system sharing several molecules with the vascular system, having a potentiality for angiogenesis, mesenchymal nature more than other tumors (Braeuer et al., 2013).

**FIGURE 8:**
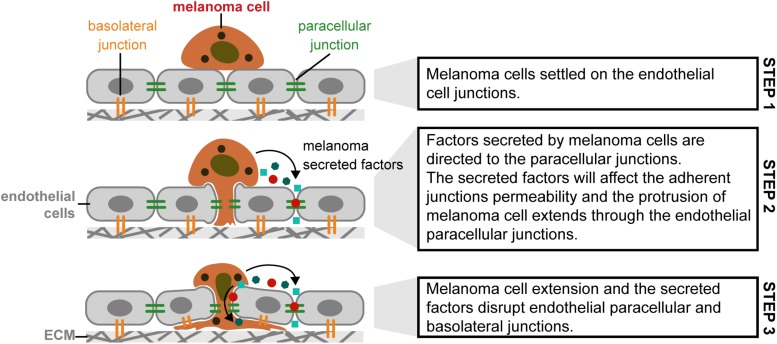
Paracellular migration mechanism used by melanoma cells in metastization of brain tissue, through the cerebral endothelial cells. (Step 1) Melanoma cells settled on the endothelial cell junctions; (Step 2) Factors secreted by melanoma cells are directed to the paracellular junctions. The secreted factors will affect the adherent junctions permeability and the protrusion of melanoma cell extends through the endothelial paracellular junctions; (Step 3) Melanoma cell extension and the secreted factors disrupt endothelial paracellular and basolateral junctions [Based in Anchan et al. (2019), Herman et al. (2019)].

The extravasation process, in melanoma, can be classified as paracellular and transcellular. The paracellular type is based on cell adhesion molecules (like CD44 and others). In transcellular extravasation, cancer cells can exit the circulatory system and penetrate through endothelial cells, not between endothelial cells (Lee et al., 2019). Regarding the metastization to the brain, Central Nervous System (CNS) has no lymphatic system, being the blood stream and the Blood-Brain Barrier (BBB) the pathway to melanoma reach the brain. Fazakas et al. (2011) investigated this process *in vitro* using cerebral endothelial cells (CECs), the A2058 metastatic lymph node and B16/F10 melanoma cell lines, respectively. Melanoma cells adhere to confluent brain endothelial cells. Melanoma cells migrate through the endothelial monolayer, continuing its movement beneath the endothelial cell layer. Melanoma cells, in contact with the brain endothelial cells, disrupted the TJ and AJ of CECs and used partially the paracellular transmigration pathway. During the experiment, melanoma cells produce and release large amount of secreted factors, which, if inhibited, decrease 44 to 55% of melanoma cell migration through the CECs. Figure 8 presents a possible explanation for paracellular migration of melanoma cells through the endothelial monolayer (Fazakas et al., 2011). In terms of forces involved in this process refer that this process in not totally understood and to our knowledge there is no literature related to this mechanism that could help to understand how the cells behave. In terms of biophysics, more work should be performed to understand the role of the forces and contractibility in the hypothesis proposed.

Melanoma cells may attach in clusters to the brain endothelial monolayer, through N-cadherin mediated junctions (Herman et al., 2019).

Some studies about melanoma transmigration were performed using human lung microvascular endothelial cells, in which was found that melanoma cells used the transcellular migration pathway (Qi et al., 2005). To our knowledge there are no studies regarding the migration (paracellular or transcellular), in liver metastization. It seems, based on literature, that both pathways can be used, being different if the metastasis achieve the brain or the lungs. In terms of driven forces in both processes, also to the best of the authors knowledge, there is no literature related to biophysical processes; more investigation should be performed in this field. Huang et al. (2018) referred that CD44 ligand-receptor interactions is also involved in cell migration and tumor cell metastization. High levels of CD44 expression will be expressed in a poor diagnosis for melanoma patients when treated with decarbazine (DTIC).

AFM force spectroscopy was used to map CD44-coupled surface receptors of melanoma cells. The effect of DTIC treatment was assessed by the dynamic binding strength and ligand free energy landscape. The unbinding force between CD44 ligand and its receptor, even when the CD44 nanodomains were reduced significantly, were not affected by the DTIC. Meanwhile, DTIC did affect the kinetic and thermodynamic interactions of the CD44 ligand–receptor, presenting greater dissociation rate, lower affinity, lower binding free energy, and a narrower energy valley for the free-energy landscape. The results demonstrated DTIC affects, but not completely inhibits, the binding of CD44 ligand to membrane receptors, suggesting the reason for the poor prognosis associated with DTIC treatment of melanoma. Therefore, AFM gives thermodynamic and kinetic insights into the effect of DTIC on the CD44 ligand-binding process (Hj et al., 2017). Bobrowska et al. (2019) also demonstrated that vertical growth phase melanoma cells showed surface biochemical and biomechanical changes in parallel, which could be used as a fingerprint of melanoma progression (Bobrowska et al., 2019).

Nikolovska et al. (2017) mentioned that when uronyl-2-O sulfotransferase (Ust) is knocked down in human melanoma cells, the amount of Ust protein is reduced, as well as adhesion and migration of melanoma cells (Nikolovska et al., 2017). Adhesion, spreading and migration of some melanoma cells depend on the presence of collagen. Type IV collagen has a complex molecular basis which is partially dependent on RGD-related sequences (Chelberg et al., 1989). Some integrins are also linked to the human melanoma progression such as the αvβ3 integrin. The highly metastatic K1735M2 murine cells expressed αvβ3 integrin, while the poorly murine metastatic K1735C23 cells did not present the αvβ3 integrin (Li et al., 2001).

Sarna et al. (2014) also found that cell melanoma pigmentation interfere in cell transmigration *in vitro*. The transmigration efficiency decreased with the increase of the apparent Young’s modulus, being proportional to the number of melanin granules present in the melanosomes inside the cell (Sarna et al., 2014).

Targeting MelCTCs in personalized therapy using markers found in cells isolated from patients’ blood stream, appears to be a promising way of preventing metastasis. The identification of MelCTCs in patient blood samples during the first year after surgical tumor removal, has an 83% correlation with the recurrence of disease after 8 months MelCTCs detection. The detection of MelCTCs in blood samples in the early stages (0-I) of melanoma development is indicative of patients with a high risk of developing metastasis (Mocellin et al., 2006). The endothelium has a potential molecular target that can be used in order to block its interactions with melanoma cell molecules; this way invasion could be prevented. Targeting MelCTCs for personalized therapy using markers found in cells isolated from patients’ blood can be a promising way of metastasis prevention (Mocellin et al., 2006).

## Melanoma: Future Perspectives

Several biophysical mechanisms, which are responsible for cell motility, invasion, adhesion, metastization, and progression in melanoma, were described in this review. It was shown that keratinocytes are an important factor in melanocyte’s control. If this control is hampered, it will lead to retraction of melanocyte’s dendrites and promotion of the movement of melanocytes through the basement membrane. Other consequences of dendrite retraction are a hindered transfer of melanosomes and melanin, not being performed by the keratinocytes anymore. Melanoma cells are characterized by the disruption of cell-cell adhesion molecules between melanocytes and keratinocytes. The internal forces generated by keratin filaments drive melanoma cell motility, like in other cancer cells. For cell motility, progression and migration, shear stress forces influence the circulation of the cancer cells contributing to the metastization in other organs. In terms of therapeutic strategies, we must invest in the development of therapies targeting disruptions between keratinocytes-keratinocytes and/or between melanocytes-keratinocytes, and the retraction of melanocyte’s dendrites. Force spectroscopy studies of adhesion molecules in adherent cells will contribute to define therapeutic strategies based on the quantification of binding forces, the energy landscape and dissociation constants of those adhesion molecules. Hampering these adhesion processes is intensively linked with the relation established by the ECM, cytoskeleton and cell nucleus. The forces produced by the tumor mass causes an increased pressure on healthy cells, which will activate several signaling pathways (mechanosensing vs. mechanotransduction), and thus transmitting these forces through the ECM, the cytoskeleton and the nucleus, causing several abnormalities in these structures, which will eventually lead to melanoma cancer progression. Keratins can be used as diagnostic biomarkers.

It was also highlighted that melanoma has a great potential of progression and metastization, and a special note was given to cancer-derived fibroblasts, since the identification of specific molecules is difficult, due to the existence of several fibroblast subpopulations. It is very important to identify the molecules participating in these pathways and quantify the forces involved, in order to understand how metastasis are formed and maintained. This also leads to the resistance of cancer to conventional treatments like radiotherapy and chemotherapy, once the specific genes expressed in this cancer, are highly mutated. These two conventional treatments affect not only tumor cells but also healthy cells. They showed fast results but harmful side effects for healthy cells. Although immunotherapy is not directed to the tumor cells themselves, reinforces the immunological system based in antibodies, vaccines and adoptive therapies. This treatment can be personalized and combined with the chemotherapy (biochemotherapy), depending on the stage of disease and the mutations on specific genes. Researchers are trying to develop biological treatments to target specific oncogenes in cancerous cells. Occasionally, some patients do not respond to immunotherapy due to the instability of cancer cells. A possible way to overcome this problem is to target some stable and not mutated cells existing on the stroma like fibroblasts, endothelial cells, immune cells and activated fibroblasts. Another strategy could be derived from a detailed knowledge of the different subtypes of fibroblasts in order to target specific molecules. The development of transfection molecules, which could reach the cell nucleus, avoiding the expression of genes, causing mutations or changes in lamins, could be another strategy. Gene therapy approaches, that inhibit the BRAF gene, could also be a possible option. As shown in this review the investigation of melanoma using different approaches and techniques, bringing together biologists, physicists, biochemists and medical specialists, is very important to deepen our knowledge and to develop novel drugs and treatments for melanoma cancer.

## Author Contributions

MMB conceived, designed, and wrote the manuscript. MR revised the manuscript, making substantial, direct and intellectual contribution to the work, and approved it for publication. SRS helped MMB to define the subject, gave intellectual contributions, revised the manuscript and approved it for publication. PLG approved the manuscript for publication.

## Conflict of Interest

The authors declare that the research was conducted in the absence of any commercial or financial relationships that could be construed as a potential conflict of interest.
